# Comparative Potential of Zinc Sulfate, L-Carnitine, Lycopene, and Coenzyme Q10 on Cadmium-Induced Male Infertility

**DOI:** 10.1155/2022/6266613

**Published:** 2022-06-30

**Authors:** Ayesha Iftikhar, Muhammad Furqan Akhtar, Ammara Saleem, Amjad Riaz, Mehrukh Zehravi, Md. Habibur Rahman, Ghulam Md Ashraf

**Affiliations:** ^1^Riphah Institute of Pharmaceutical Sciences, Riphah International University, Lahore Campus, Lahore, Pakistan; ^2^Department of Pharmacology, Faculty of Pharmaceutical Sciences, Government College University Faisalabad, Faisalabad, Pakistan; ^3^Department of Thriogenology, University of Veterinary and Animal Science, Lahore, Pakistan; ^4^Department of Clinical Pharmacy Girls Section, Prince Sattam Bin Abdulaziz University, Al-Kharj, Saudi Arabia; ^5^Department of Pharmacy, Southeast University, Banani, Dhaka 1213, Bangladesh; ^6^Department of Global Medical Science, Yonsei University Wonju College of Medicine, Yonsei University, Wonju, Gangwon-do 26426, Republic of Korea; ^7^Preclinical Research Unit, King Fahd Medical Research Center, King Abdulaziz University, Jeddah, Saudi Arabia; ^8^Department of Medical Laboratory Technology, Faculty of Applied Medical Sciences, King Abdulaziz University, Jeddah, Saudi Arabia

## Abstract

The human exposure to toxic chemicals and heavy metals is one of the main predisposing factors contributing to male infertility. Acute exposure to cadmium chloride results in testicular damage and infertility. The purpose of the present study was to investigate and compare the curative effect of coenzyme Q10 (CoQ10), lycopene, L-carnitine (LC), and zinc sulfate against the cadmium-induced infertility in male Wistar rats. Cadmium chloride (0.4 mg/kg/day) was orally administered to rats for three consecutive days. Then, oral administration of different treatments (i.e., LC 100 mg/kg, CoQ10 20 mg/kg, lycopene 4 mg/kg, zinc sulfate 6 mg/kg, and a combination LC-CoQ10 at 500/50 mg/kg) was carried out for 30 days. The impact of different treatments on semen parameters, such as sperm count and motility, testicular antioxidants, and serum testosterone, was determined. Furthermore, the morphology of epididymis sperms and histopathology of rat testes were also assessed. Cadmium exposure decreased the sperm count, progressive sperm motility, testosterone, superoxide dismutase (SOD), and catalase and reduced glutathione (GSH). It also caused banana sperm tail, bent sperm head, vacuolization of seminiferous tubules, and oligospermia in rat testes. All treatments with nutraceuticals improved sperm count, sperm morphology, serum testosterone, vacuolization of seminiferous tubules, and oligospermia in diseased rats. Treatment with lycopene, LC, and LC-CoQ10 improved progressive sperm motility and other parameters and increased SOD, GSH, and CAT in the rat testes. CoQ10 also increased SOD activity in rat testes' tissue homogenates. It is concluded from the current study that all nutraceuticals partially improved reproductive toxicity of cadmium. The administration of lycopene and a high-dose combination of LC-CoQ10 were more efficacious in treating cadmium-induced infertility than other treatments. Treatment of cadmium-exposed rats with lycopene, LC, CoQ10, and LC-CoQ10 improved sperm count and motility through reduction of testicular oxidative stress and improving serum testosterone.

## 1. Introduction

It is estimated that the infertility affects about 15% couples globally and this frequency is gradually increasing [1]. The male infertility is traditionally caused by varicocele, cryptorchidism, infections, obstructive lesions, trauma, tumors, and hormonal and environmental factors. Oxidative stress due to excessive reactive oxygen species (ROS) raises numerous problems related to male infertility, like sperm damage and sperm deformity [2]. The pathophysiological origins of infertility include hypogonadotropic hypogonadism (HH), coital disorders, erectile dysfunction, and ejaculation disorder. In male HH, testes fail to produce androgen and sperms, resulting in congenital or acquired diseases, affecting hypothalamus and/or the pituitary gland. Spermatogenesis and androgen production are maintained and induced in HH by exogenous regulation of gonadotropins, permitting natural fertility in several cases [3]. Clomiphene citrate and selective estrogen receptor modulator (SERM) are mainly used to treat HH [4–6].

Several heavy metals cause the male infertility, such as cadmium, lead, and mercury. Acute cadmium poisoning can lead to hepatic and testicular injury. Ototoxicity, severe testicular hemorrhage, edema, and necrosis have resulted from chronic exposure to cadmium. Cadmium 21 is the analogue of cadmium, which is toxic to humans and animals [7]. Cadmium absorption occurs in human by ingestion or inhalation of the food containing cadmium. Mostly, cadmium present in terrestrial foods is absorbed into the body. Cadmium is used in batteries, metal plating, pigments, and in plastics and alloy industries. Human exposure to cadmium occurs ecologically or by occupational ways. Ingestion of food, drinking water, contaminated soil and dust, cigarette, smoking, and dietary consumption are the main causes of cadmium exposure to human [8]. Cadmium inactivates the enzyme containing sulfhydryl groups. It also causes the uncoupling of oxidative phosphorylation in mitochondria of the cell. Moreover, it competes with other metals (e.g., Zn and selenium) for attachment to metalloenzymes and with calcium for binding sites (e.g., calmodulin on regulatory proteins). Cadmium develops the oxidative stress in the different organs by acute poisoning and causes severe degenerative changes in most tissues leading to osteomalacia, hepatotoxicity, renal toxicity, neurotoxicity, infertility, and cancer [9].

Oxidative stress is implicated in several human diseases including but not limited to atherosclerosis, cancer, diabetes, rheumatoid arthritis, inflammatory bowel disease, Parkinson's disease, and infertility [10]. Although small amount of ROS is beneficial for fertilization, the excessive amounts of ROS and nitric oxide (NO) promote capacitation and acrosomal reaction, thus adversely affecting spermatogenesis. Even the lipid peroxidation resulting from low level of ROS causes the plasma membrane modification and facilitates the adhesion of sperm-oocyte. These prooxidants are then shifted to the semen and vaginal secretions, which ultimately cause the infertility [11]. Morphologically abnormal spermatozoa are produced by the ROS in the infertile men having low capacity of the antioxidants and less sperm motility. In the seminal plasma of asthenozoospermic men, the ROS activity is associated with the chain-breaking antioxidants [12]. Dietary and endogenous antioxidants including low level of chain-breaking antioxidants (CBA) are indicated to treat reduced spermatogenesis and testicular stress [13].

Coenzyme Q10 (CoQ10) protects from thrombolysis, congestive heart failure (CHF), essential hypertension, renovascular hypertension, and ventricular arrhythmia in addition to stroke, retinopathy, and muscular dystrophy. Moreover, CoQ10 is widely distributed to different body organs and exhibits a long half-life [14]. Lycopene belongs to carotenoids, which are the main sources of vitamin A. Lycopene possesses the antioxidant properties and is naturally found in fruits and vegetables. Lycopene inhibits various human cancer cells, prevents DNA damage, and is more potent than *α*- and *ß*-carotenes [15].

L-Carnitine (LC) belongs to carnitines, which are highly polar compounds, broadly distributed in nature. Within the male genital tract, carnitines are concentrated in the epididymis and spermatozoa [12]. Carnitines are also frequently used in the treatment of male infertility, chronic renal failure, and erythropoietin resistant anemia [16]. Zinc (Zn) is an essential trace element that acts as a cofactor involved in deoxyribonucleic acid (DNA) profiling and protein synthesis. Furthermore, Zn has also antiapoptotic and antioxidant properties. Zn is useful in the treatment of asthenozoospermic male infertility. It improves the sperm integrity, conception, and pregnancy rates. Zn also plays a pivotal role in lipid metabolic flexibility and stabilization of sperm membrane [17]. Lack of Zn is related to the compromised sperm functioning and enhancement of oxidative stress in seminal plasma. Moreover, Zn is also important in the regulation of various processes, for example, capacitation and the acrosome reaction of sperm for conception and embryonic implantation [18]. The investigation reason behind this study was to assess and compare the efficacy of lycopene, LC, ZnSO_4_, and CoQ10 against cadmium-induced infertility in male rats.

## 2. Materials and Methods

Diethyl ether, phosphate buffer solution (PBS), paraformaldehyde, ethanol, xylene, paraffin, hematoxylin, 10% tricyclic acid (TCA), dithiobis-2-nitro benzoic acid (DTNB), pyrogallol solution, potassium phosphate, hydrogen peroxide (H_2_O_2_), and cadmium chloride were obtained from Sigma Aldrich (Germany). LC and zinc sulfate (ZnSO_4_) were obtained from Selmore Pharmaceuticals (Pakistan). CoQ10 and lycopene were acquired from Bionext Pharmaceuticals, Pakistan. Eosin-Nigrosin stain was obtained from Hitech Specialties Solutions, India. Centrifuge (800 D–4000 rpm), microtome, compound microscope (Germany), Eppendorf tubes, UV-visible spectrophotometer (UV-1601, Shimadzu), and computer-assisted sperm analysis (CASA) system (Minitube®, Germany) were used in the study.

### 2.1. Experiment Design

Seventy male Wistar rats of 10 weeks old and weighing 150 ± 20 g were used in the study. The animals were acquired from Riphah Institute of Veterinary Sciences, Lahore, Pakistan, and then acclimatized for two weeks in well-ventilated animal house of RIPS, Lahore, Pakistan. The animal study was approved and conducted according to the guidelines of the Institutional Ethical Committee of Riphah International University (Reference no. REC/RIPS-LHR/2017044). Animals were kept at a temperature of 26 to 30°C and humidity of 30 to 70%, maintained according to the National Institute of Health (NIH) guidelines. These rats were randomly divided into seven groups, with each group comprising of ten animals. All animal groups were separately placed in steel cages. To demonstrate the curative effect of different nutraceuticals against male infertility, the LC, CoQ10, lycopene, and ZnSO_4_ were orally given to rats for 30 days previously treated with cadmium chloride. Afterwards, the sperm, biochemical, hormonal, and histological parameters were determined. Different treatments given to various groups are given in Table 1.

Cadmium was administered orally to rats for three times on consecutive days. Then, different treatments with nutraceuticals were orally administered for 30 days. 24 h after administration of last doses, the rats were anesthetized with diethyl ether, the blood samples were collected from heart puncture, and their peritoneal cavities were opened through a low transverse incision to remove the testes of rats from the control and treatment groups immediately. The blood taken in plain tubes was centrifuged (3000 rpm) at 4°C for 10 min to prepare the serum from each animal and stored it at −20°C until assayed. The harvested testes specimens were preserved in paraformaldehyde for histological analysis [23].

### 2.2. Epididymis Sperm Count, Viability, and Motility

Determination of the motility was done by the CASA system. The contralateral epididymis was transferred to another petri dish containing PBS followed by the diffusion method in which sperms diffused from epididymis tubes. The layers of connective tissue were removed at the juncture between the proximal and distal regions of the cauda epididymis using Jewelers forceps. Approximately within the 30 S, the study samples were picked up to avoid variations that occur during late samples. The sperm viability was determined by staining with Eosin-Nigrosin [24]. Sperm motility parameters such as total motility, progressive motility of sperms (PMS), progressive fast motility (PFM), progressive slow motility (PSM), progressive circular motility (PCM), and linear motility of sperms (LMS) were determined.

### 2.3. Morphological Studies

Abnormal spermatozoa in sperm cells were evaluated after obtaining from the cauda epididymis and calculated according to the Wyrobk method by counting and identifying 1000 sperm heads in three samples per animal. The abnormalities observed in spermatozoa were categorized into four groups according to their frequency of appearance.

Spermatozoa were observed for flagellum abnormalities such as dual, bifid, tangled or wrong insertion of the flagellum, abnormalities in head morphology such as macro- or microcephaly; acephalous, strange head shape, and alterations in acrosome [25].

### 2.4. Histological Studies

Tissues of testes were preserved in a solution of 4% paraformaldehyde in 0.1 M phosphate buffer. Then, tissues were detached, dried by ethanol and then sanitized with xylene, and lastly embedded in paraffin wax. Microtome was used for preparing tissue section of 5 *μ*m thickness. Hematoxylin-eosin staining was used for countermarking of tissues, which were observed under light microscope for examination and photomicrographs [26].

### 2.5. Hormonal Assay

The serum obtained from the collected blood was used to determine the hormone profile. Testosterone level was measured using commercially available immunoassay enzyme linked immunoassay (Rocky Mountain Diagnostics, Inc., USA, according to manufacturer's specs).

### 2.6. In Vivo Antioxidant Activity

Enzymatic antioxidants such as glutathione (GSH), catalase (CAT), and superoxide dismutase (SOD) were assessed in rat testes by the methods described previously. To prepare tissue homogenate, harvested testicular tissues were washed with ice-cold normal saline; 1 g tissue was minced with 2-3 ml PBS via tissue homogenizer and then diluted with PBS to prepare 10% w/v tissue homogenate. The resultant mixture was centrifuged at 4000 rpm for 20 min and the supernatant was drawn into a sterile tube and oxidative stress markers were determined immediately. Moreover, protein content in testicular tissue homogenate was determined by Lowry methods [27].

### 2.7. GSH Level

For GSH determination, a previously described method was followed [27]. A 1 ml of tissue homogenate supernatant was taken and added to 1 ml of 10% TCA, 4 ml of sodium phosphate solution, and 0.5 ml DTNB. The absorbance of resultant solution was taken at 412 nm with UV-Vis spectrophotometer.

### 2.8. SOD Activity

An earlier described method was used to determine SOD activity with little modifications [10]. A 100 *μ*l tissue homogenate supernatant was added to 2.8 ml PBS (pH of 7.4) and 100 *μ*l pyrogallol solution. The absorbance of the mixture was taken at 325 nm. One unit of SOD was described as the amount of enzyme responsible for 50% inhibition of pyrogallol autoxidation per ml of the assay solution.

### 2.9. CAT Activity

The activity of CAT was assessed by H_2_O_2_ method. A 50 *μ*l tissue homogenate was added to 1.95 ml potassium phosphate solution and (50 mM) 1 ml of H_2_O_2_. Change in absorbance per min was determined at 240 nm to estimate CAT activity [28].

### 2.10. Statistical Analysis

The obtained results were tabulated and presented as mean ± standard deviation (SD) and evaluated by GraphPad Prism software version 7.01 for one-way analysis of variance (ANOVA) followed by a post hoc test. Bonferroni's multiple comparison tests showed the significance level of *P* < 0.001, *P* < 0.01, and *P* < 0.05.

## 3. Results

To demonstrate the curative effect of different nutraceuticals against male infertility, the LC, CoQ10, lycopene, and ZnSO_4_ were orally given to rats for 30 days previously treated with cadmium chloride. Afterwards, the sperm, biochemical, hormonal, and histological parameters were determined.

### 3.1. Epididymis Sperm Count

**Figure 1:**
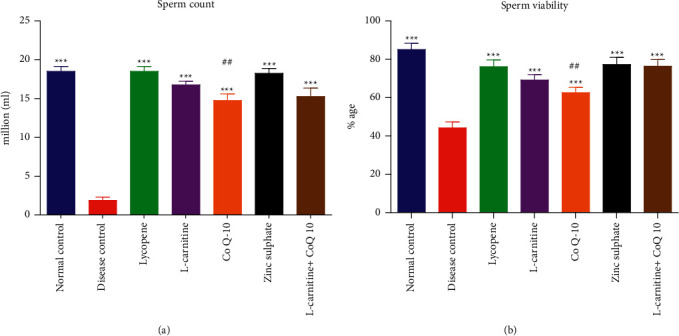
Effect of lycopene, L-carnitine, coenzyme Q10, and zinc sulfate on sperm count and viability of cadmium-exposed rats. Results presented as mean ± S.D. (*n* = 10). ^∗∗∗^and ^##^showed statistically significant as compared to disease control and normal control at *P*< 0.001 and *P*<0.01 respectively.

After 30 days of dosing, the sperm count was observed in different groups, which indicated that the disease control group had shown significantly reduced sperm count (1.76 ± 0.67 million/ml) as compared to the normal control rats (18.55 ± 0.95 million/ml). Treatment with different nutraceuticals significantly improved the sperm count in cadmium-treated rats. The sperm count in lycopene-treated group (18.33 ± 0.64 million/ml) was the highest among all treated groups which was followed by ZnSO_4_ (17.95 ± 0.47 million/ml), LC (16.77 ± 0.74 million/ml), LC-CoQ10 (15.24 ± 0.64 million/ml), and CoQ10 (14.58 ± 0.85 million/ml) treated groups. Moreover, the sperm count in all nutraceutical treated rats resulted in a rise as compared to that of normal control rats except CoQ10 treated rats, which showed statistically reduced sperm count in comparison to the normal control group. The effect of nutraceuticals on sperm viability in cadmium exposed rats was also significant. All nutraceuticals increased the sperm viability in diseased rats. The effect of different nutraceuticals on sperm count and viability of cadmium exposed rats is shown in Figure 1.

Results are presented as mean ± SD (*n* = 10). ^*∗∗∗*^ and ## showed statistical significance as compared to disease control and normal control at *P* < 0.001 and *P* < 0.01, respectively.

### 3.2. Motility of Sperms

**Figure 2:**
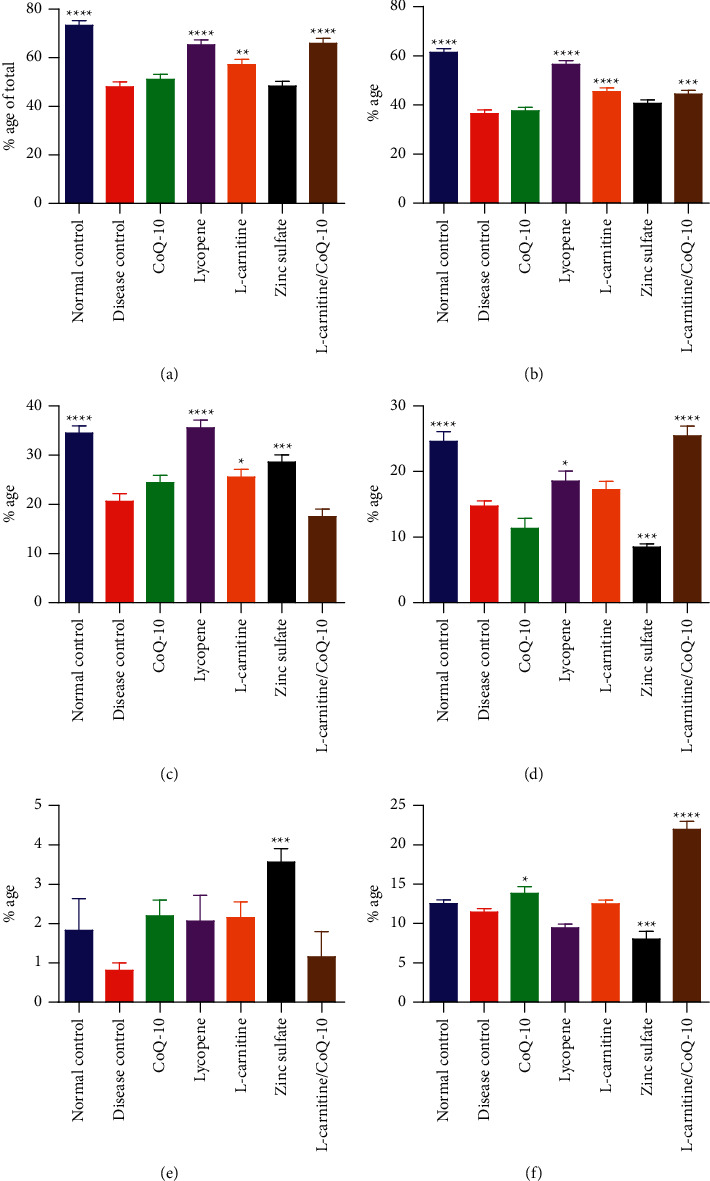
Effect of lycopene, L-carnitine, coenzyme Q10, and zinc sulfate on sperm motility of cadmium-exposed rats. Results presented as mean ± S.D. (*n* = 10). ^∗∗∗∗^, ^∗∗∗^, ^∗∗^ and ^∗^ showed statistically significant as compared to diseased control at *P*< 0.0001, 0.001, 0.01 and 0.05 respectively. (a) Total motility. (b) Progressive motility. (c) Progressive fast motility. (d) Progressive slow motility. (e) Progressive circular motility. (f) Local motility.

The CASA analysis revealed the motility of sperms in the cadmium-exposed rats. Treatment with nutraceuticals had variable effect on sperm motility parameters such as total motility, PMS, PFM, PSM, PCM, and LMS. The effect of various treatments on cadmium-induced infertility in rats is shown in Figure 2.

It was found that the total motile sperms in the disease control group were significantly declined (*P* < 0.0001) in comparison to the normal control rats. Administration of lycopene, LC, and LC-CoQ10 increased the percentage of motile sperms in cadmium-treated rats to varying degree. Lycopene and LC-CoQ10 had the most pronounced positive effect on sperm motility followed by the LC.

Administration of cadmium significantly reduced the percentage of PMS in disease control rats as compared to normal control rats. It was revealed that the administration of lycopene, LC, and LC-CoQ10 had a profound positive effect on PMS in rats preexposed to cadmium. Treatment with lycopene had the most prominent effect on PMS in rats while LC and LC-CoQ10 had a comparative effect on PMS of cadmium-exposed rats.

It was found that cadmium exposure in rats significantly decreased the percentage of PFM sperms as compared to normal rats. Administration of CoQ10 and LC-CoQ10 failed to bring about any significant increase in PFM in cadmium-exposed rats. However, treatment with lycopene, LC, and ZnSO_4_ significantly improved the percentage of PFM in rats. Administration of lycopene exhibited the most pronounced increase of PFM in cadmium exposed rats as shown in Figure 2. Cadmium exposure also significantly decreased PSM in treated rats in contrast to the normal control group. Treatment with LC-CoQ10 exhibited the most significant increase in PSM in comparison to the disease control group followed by lycopene. However, the administration of CoQ10, LC, and ZnSO_4_ did not cause any increase in the PSM of cadmium-exposed rats, as shown in Figure 2.

It was evident that the exposure to cadmium had not brought about any statistically significant decline in the percentage of PCM in the disease control group. Administration of ZnSO_4_ resulted in a significant (*P* < 0.001) increase in PCM in contrast to disease control. However, the treatments with lycopene, CoQ10, LC, and LC-CoQ10 had insignificantly affected the PCM induced by cadmium. Furthermore, cadmium exposure did not cause any significant decline in the percentage of LMS in rats. However, administration of CoQ10 and LC-CoQ10 resulted in a statistically significant increase in the LMS of cadmium-exposed rats, as shown in Figure 2.

Results are presented as mean ± SD (*n* = 10). ^*∗∗∗∗*^, ^*∗∗∗*^, ^*∗∗*^ and ^*∗*^ showed statistical significance as compared to disease control at *P* < 0.0001, 0.001, 0.01, and 0.05, respectively.

### 3.3. Sperm Morphology

**Figure 3:**
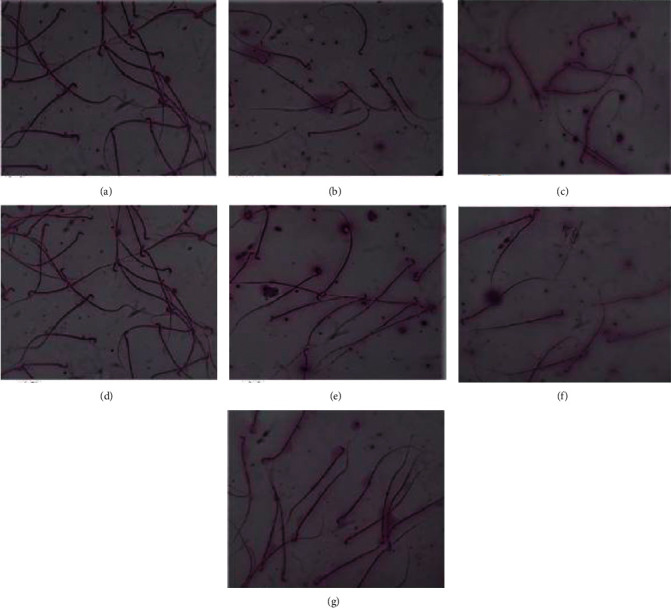
Effect of lycopene, L-carnitine, coenzyme Q10, and zinc sulfate on sperm morphology of cadmium-exposed rats at 100x resolution. (a) Normal control. (b) Disease control. (c) L-Carnitine/coenzyme Q10 treated. (d) Lycopene treated. (e) L-carnitine treated. (f) Zinc sulfate treated. (g) Coenzyme Q10.

In morphological studies, general observation was made to determine various abnormalities of spermatozoa in all groups. The abnormalities appeared in the flagellum and head of rat sperm exposed to cadmium. Bent sperm tail and banana head were observed in several sperms of the disease control group. Such abnormalities were not evident in groups treated with lycopene, LC, ZnSO_4_, CoQ10, and LC-CoQ10. The effect of different treatments on sperm morphology is shown in Figure 3.

### 3.4. Effect on Testosterone Level

**Figure 4:**
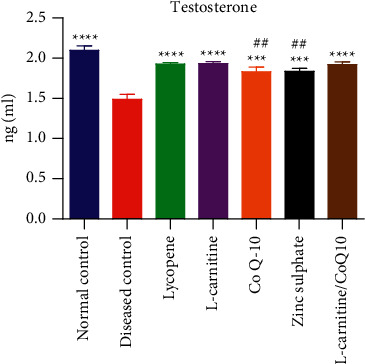
Effect of lycopene, L-carnitine, coenzyme Q10, and zinc sulfate on serum testosterone of cadmium-exposed rats.

The serum level of testosterone in normal control group (2.39 ± 0.507 ng/ml) was higher as compared to cadmium-exposed rats (1.46 ± 0.096 ng/ml). All nutraceuticals significantly improved the level of serum testosterone in cadmium-exposed rats. The treatment with lycopene, LC, and LC-CoQ10 resulted in the most pronounced effect on serum testosterone level in cadmium exposed rats and were significantly comparable to a normal control group. However, the treatment with CoQ10 and zinc sulfate resulted in lower serum testosterone level as compared to normal control rats. The effect of different nutraceuticals on the serum testosterone level of cadmium exposed rats is shown in Figure 4.

Results are presented as mean ± SD (*n* = 10). ^*∗∗∗∗*^ and ^*∗∗∗*^ showed statistical significance as compared to the diseased group at *P* < 0.0001 and 0.001, respectively, while ## showed statistical significance to normal control at *P* < 0.01.

### 3.5. Oxidative Stress Parameters in Rat Testes

Administration of cadmium was followed by treatment with different nutraceuticals and then rat testes' tissue homogenates (10% w/v) were tested for antioxidant parameters, such as SOD, CAT, and GSH.

### 3.6. SOD Activity

**Figure 5:**
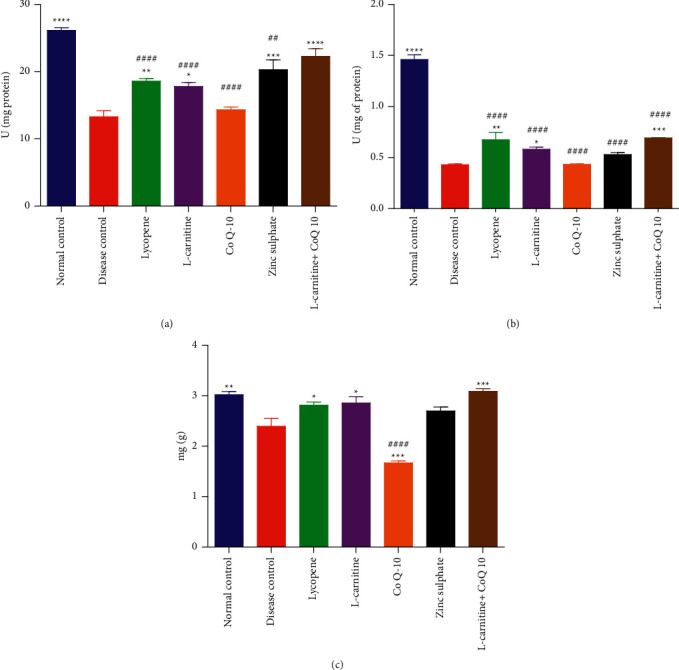
Effect of lycopene, L-carnitine, coenzyme Q10, and zinc sulfate on oxidative stress in testicles of cadmium-exposed rats. Results presented as mean ± S.D. (*n* = 10). ^∗∗∗∗^, ^∗∗∗^, ^∗∗^ and ^∗^ showed statistically significant as compared to disease control at *P*< 0.0001, 0.001, 0.01 and 0.05 respectively, while ^####^ and ^##^ showed statistically significant compared to normal control at *P*<0.0001 and 0.01 respectively. (a) Superoxide dismutase. (b) Catalase. (c) Reduced glutathione.

In the current study, the activity of SOD was found to be significantly higher in normal control group (26.13 ± 0.78 U/mg of protein) as compared to the disease control group (13.29 ± 1.78 U/mg of protein). Treatment with LC-CoQ10 (22.28 ± 2.255 U/mg of protein) showed the highest SOD activity among all nutraceuticals, which was statistically comparable to the normal control group. Treatment with lycopene and LC also exhibited statistically higher SOD activity than the disease control group. The activity of SOD exhibited by LC-CoQ10 was insignificantly different from the normal control group, as shown in Figure 5(a).

Results are presented as mean ± SD (*n* = 10). ^*∗∗∗∗*^, ^*∗∗∗*^, ^*∗∗*^, and ^*∗*^ showed statistical significance as compared to disease control at *P* < 0.0001, 0.001, 0.01, and 0.05, respectively, while #### and ## showed statistical significance compared to normal control at *P* < 0.0001 and 0.01, respectively.

### 3.7. CAT Activity

It was found that the activity of CAT in normal control group (1.46 ± 0.096 U/mg of protein) was statistically higher as compared to the disease control group (0.42 ± 0.013 U/mg of protein). The activities of CAT in lycopene, LC, and LC-CoQ10 groups were significantly elevated than the disease control group. However, LC, lycopene, and LC-CoQ10 failed to normalize the CAT activity. The treatment with ZonSO_4_ and CoQ10 did not improve the CAT activity in comparison to the disease control group as shown in Figure 5.

### 3.8. GSH Level

It was found that the GSH content in the normal control group (3.02 ± 0.123 nmol/ml) was significantly higher as compared to disease control group (2.39 ± 0.318 mg/g). Administration of lycopene, LC, and LC-CoQ10 significantly increased GSH in diseased rats. The effect of ZnSO_4_ on GSH was negligible in comparison with the disease control group. Moreover, the treatment with CoQ10 significantly reduced the GSH content in cadmium-exposed rats. The effect of treatment with lycopene, L-carnitine, CoQ10, and ZnSO_4_ on cadmium-induced reduction in GSH content in rat testicular tissue homogenate is as shown in Figure 5.

### 3.9. Histological Studies

**Figure 6:**
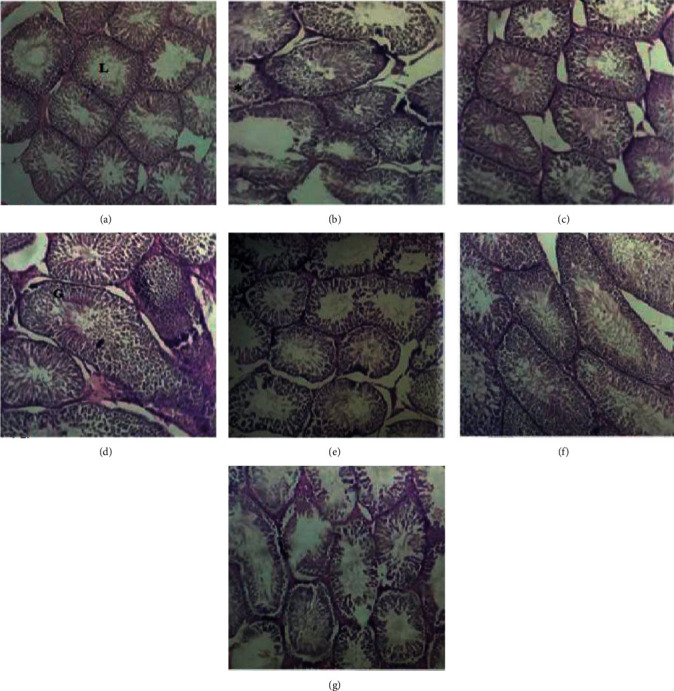
Histological photomicrographs of cadmium-exposed rat testes treated with zinc chloride, lycopene, coenzyme Q10, and L-carnitine. (a) Normal control group. (b) Disease control group. (c) Zinc chloride. (d) Coenzyme Q10. (e) Lycopene. (f) L-Carnitine. (g) Coenzyme Q10 and L-carnitine treated group, whereas “L” showed seminal tubule, “G” germinal epithelium, and *∗* shows vacuole formation.

Photomicrograph of testicular histology of the normal control rats showed intact and normal structure germinal epithelium. However, treatment with cadmium chloride resulted in vacuolization in germinal epithelial cells and oligospermia in the testes of diseased rats. The fissures were observed clearly, and the seminiferous epithelium was incomplete. Treatment with lycopene, CoQ10, LC, ZnSO_4_, and LC-CoQ10 resulted in amelioration of cadmium toxicity. All treatments with CoQ10, LC, ZnSO_4_, and LC-CoQ10 showed only partial improvement in testicular lesion caused by cadmium chloride. The damage of histological structures in the LC-CoQ10 and L-carnitine treated group was even worse than that in other nutraceutical treatment groups. The effect of treatment with lycopene, L-carnitine, CoQ10, and ZnSO_4_ on cadmium-induced histological damage to rat testicles is shown in Figure 6.

## 4. Discussion

Several drugs, heavy metals, and environmental chemicals have toxic effects on human reproductive system. Human and animals are exposed to cadmium through occupational activity, environment, dust, water, smoking, and food items [29]. Male infertility is one of the most important factors that causes the disturbances in everyday life. Oxidative stress is the main reason for male infertility along with hormonal imbalance. The current study investigated the effect of CdCl_2_ on infertility in male rats and potential effects of lycopene, CoQ10, LC, ZnSO_4_, and LC-CoQ10 on CdCl_2_-induced infertility through assessing semen parameters, measuring testosterone and testicular antioxidants level such as SOD, CAT, and GSH, as well as evaluating testicular histology.

Cadmium is found to cause male infertility through reduction of the weights of testes and epididymis and accumulation in rat testes to exhibit apoptosis, decreased serum testosterone, and elevated oxidative stress and cellular glucose uptake [30]. In the present study, semen parameters such as sperm count and motility were decreased in Wistar rats after exposure to cadmium. These findings are in line with previous study, which showed that reduction in sperm count is associated with heavy metals, such as cadmium [31]. Treatment with lycopene, ZnSO_4_, CoQ10, and LC significantly raised sperm count. In addition to sperm count, male fertility is dependent upon the PMS, which is inversely corelated to DNA fragmentation leading to infertility [32]. As the present study demonstrated, the reduction in all sperm motility parameters of the disease control group indicated the DNA fragmentation and male infertility. Administration of lycopene, LC, and LC-CoQ10 increased PMS in comparison to the disease control rats. Administration of lycopene has previously shown that it prevented the DNA damage associated with oxidative stress in vitro in human sperms [33]. Moreover, LC has also demonstrated cryoprotective effect on human spermatozoa through prevention of DNA oxidative, which results in improved in vitro fertility [34]. Previous investigations also proved that lycopene, LC, CoQ10, and Zn had improved sperm count and motility [12]. The treatment with antioxidants showed a remarkable improvement in sperm count and viability by reduction in ROS [35].

In morphological study, spermatozoa abnormalities, such as vacuolization and oligospermia, were observed in cadmium-treated rats. Animals treated with lycopene, CoQ10, LC, and ZnSO_4_ showed no abnormal spermatozoa in contrast to the disease control group. A previous study showed that treatment of cyclophosphamide-induced infertile Sprague‐Dawley male adult rats with lycopene normalized the sperm morphology [36]. Previous studies confirm that the lycopene, LC, CoQ10, and ZnSO_4_ and CoQ10 supplementations reduced morphological anomalies in diseased or injured animals [37].

The current study also showed the remarkable inhibition in serum testosterone level by cadmium exposure. The treatments with lycopene, LC, CoQ10, and ZnSO_4_ and CoQ10 significantly raised the level of testosterone, sperm count, sperm motility, and antioxidant enzyme concentrations after acute poisoning of cadmium as compared to control group [35]. Testosterone level is an important factor in the determination of male infertility. A previous study conducted on LC showed the similar effect on serum testosterone level [20]. A study conducted on LC showed a significant increase in plasma testosterone level in male adult albino mice that were treated with cadmium chloride at dose 0.35 mg/kg [38]. Similarly, a study conducted on ZnSO_4_ in improving fertility also supported that Zn had a positive effect on testosterone level and semen quality in infertile 7-week-old Sprague Dawley rats [39]. CoQ10 also reversed the testicular damage associated with cadmium toxicity by enhancing the levels of LH and testosterone in rodents likewise the present study [39].

It is found that the reduction in the amount of glutathione and protein containing sulfhydryl group is associated with cadmium exposure, which eventually leads to increased ROS production. This enhanced production of ROS increases the lipid peroxidation, excretion of urinary lipid metabolites, DNA damage, membrane damage, altered gene expression, and apoptosis [31]. The in vivo antioxidants such as SOD, CAT, and GSH were reduced by exposure to cadmium in diseased rats. Treatment with LC, lycopene, ZnSO_4_, and LC-CoQ10 raised the activity of SOD in rat testes as compared to the disease control group. Moreover, the CAT activity and GSH content were increased by treatment with LC and lycopene and LC-CoQ10 in testicles of cadmium-exposed rats. It is previously found that LC reduced oxidative stress through reduction of malondialdehyde (MDA) and increasing GSH in lipopolysaccharide exposed rats at 500 mg/kg single dose [40]. The in vivo antioxidants (SOD, CAT, and GSH) play a pivotal role in the reduction of oxidative stress and thus decrease the chances of infertility. In case of histopathological study, disease control group showed vacuolization in germinal epithelial cells whereas the administration of LC, lycopene, CoQ10, ZnSO_4_, and LC-CoQ10 in infertile rats showed no deterioration as evident in previous study [41].

Previous study on titanium dioxide and benzopyrene-induced toxicity reported that its testicular toxicity was treated by lycopene through reduction of oxidative stress and suppressing the apoptosis to protect germinal cells [42, 43]. Furthermore, lycopene has demonstrated the anti-infertility effect against drug-induced testicular damage caused by gentamicin, cisplatin, and deltamethrin mainly through the reduction of lipid peroxidation and oxidative stress and increased production of testosterone [44]. Furthermore, lycopene was useful to improve infertility caused by coal burning related fluorosis through oxidative stress associated with Jun N-terminal kinase (JNK) and extracellular signal-regulated protein kinase (ERK) pathway [45].

The LC has also shown improvement of oligospermia against busulfan-induced oligospermia through reduction of oxidative stress [46]. Moreover, LC-ameliorated trazodone induced testicular damage through modulating autophagy, reduction of oxidative stress, and preventing inflammation [47]. LC also corrects testicular perfusion to prevent ischemic injury to testicular tissues [48]. Prevention of apoptosis is also one of the mechanisms through which LC might have demonstrated anti-infertility effect against toxicants through modulation of proapoptotic and antiapoptotic genes, such as Bcl-2, BAX, and Caspase-3 [49]. Posttreatment of diseased rats with CoQ10 failed to bring out an increase in the antioxidants such as GSH, CAT, and SOD, which is in contrast to a previous study that demonstrated the preventive effective of CoQ10 against cadmium-induced infertility had been mediated through reduction of oxidative stress. This apparent contrast might be due to preventive treatment of cadmium-exposed rats with CoQ10 in the previous study [19]. However, estimation of MDA, an important indicator of lipid peroxidation, must also be carried out in rats' testes exposed to cadmium as cadmium has been found to increase lipid peroxidation in different animal organs [50].

## 5. Conclusion

In conclusion, the present study showed that the male infertility caused by exposure to cadmium was associated with increased sperm immobility, reduction in sperm count and viability, damage to testicular histology, and increase of oxidative stress. Daily administration of LC 100 mg/kg, lycopene 4 mg/kg, ZnSO_4_ 6 mg/kg, and LC-CoQ10 500/50 mg/kg improved the sperm parameters, serum testosterone, testicular histology, and oxidative stress in male rats to varying degree. Administration of individual and combination treatment of nutraceuticals to cadmium-exposed rats improved cadmium chloride-induced toxicity through the reduction of oxidative damage and increase serum testosterone. Further studies must be carried out to evaluate the ameliorating effect of these chemicals against other endocrine-disrupting effects of cadmium. These nutraceuticals must also be evaluated individually and in combination with each other against infertility caused by heavy metals and other endocrine disruptors.

## Figures and Tables

**Table 1:** Treatment plan for study of cadmium-induced infertility in male rats.

Groups	Treatments
1	Normal control: received distilled water orally
2	Disease control: cadmium 0.4 mg/kg/day as cadmium chloride orally for three consecutive days [19]
3	Cadmium 0.4 mg/kg/day as cadmium chloride thrice + CoQ10 20 mg/kg/day [19]
4	Cadmium 0.4 mg/kg/day as cadmium chloride thrice + lycopene 4 mg/kg/day suspended in corn oil [20]
5	Cadmium 0.4 mg/kg/day as cadmium chloride thrice + L-carnitine 100 mg/kg/day [21]
6	Cadmium 0.4 mg/kg/day as cadmium chloride thrice + ZnSO_4_ 6 mg/kg/day [22]
7	Cadmium 0.4 mg/kg/day as cadmium chloride thrice + L-carnitine/CoQ10: 500/50 mg/kg/day

## Data Availability

The data used to support this study are available from the corresponding author upon request.

## References

[1] Abu-Naser S. S. (2016). Male infertility expert system diagnoses and treatment. *American Journal of Innovative Research and Applied Sciences*.

[2] Sharlip I. D., Jarow J. P., Belker A. M. (2002). Best practice policies for male infertility. *Fertility and Sterility*.

[3] Wagner H., Cheng J. W., Ko E. Y. (2018). Role of reactive oxygen species in male infertility: an updated review of literature. *Arab Journal of Urology*.

[4] Moskovic D. J., Katz D. J., Akhavan A., Park K., Mulhall J. P. (2012). Clomiphene citrate is safe and effective for long-term management of hypogonadism. *BJU International*.

[5] Resteghini C., Cavalieri S., Galbiati D. (2017). Management of tyrosine kinase inhibitors (TKI) side effects in differentiated and medullary thyroid cancer patients. *Best Practice & Research Clinical Endocrinology & Metabolism*.

[6] Leaver R. B. (2016). Male infertility: an overview of causes and treatment options. *British Journal of Nursing*.

[7] Yari A., Asadi M. H., Bahadoran H., Dashtnavard H., Imani H., Naghii M. R. (2010). Cadmium toxicity in spermatogenesis and protective effects of L-carnitine in adult male rats. *Biological Trace Element Research*.

[8] Martínez D., Grindlay G., Gras L., Mora J. (2018). Determination of cadmium and lead in wine samples by means of dispersive liquid–liquid microextraction coupled to electrothermal atomic absorption spectrometry. *Journal of Food Composition and Analysis*.

[9] Monsefi M., Alaee S., Moradshahi A., Rohani L. (2010). Cadmium‐induced infertility in male mice. *Environmental Toxicology: International Journal*.

[10] Akhtar M. F., Khan K., Saleem A., Baig M. M. F. A., Rasul A., Abdel-Daim M. M. (2021). Chemical characterization and anti-arthritic appraisal of monotheca buxifolia methanolic extract in complete freund’s adjuvant-induced arthritis in wistar rats. *Inflammopharmacology*.

[11] Makker K., Agarwal A., Sharma R. (2009). Oxidative stress & male infertility. *Indian Journal of Medical Research*.

[12] Agarwal A., Virk G., Ong C., du Plessis S. S. (2014). Effect of oxidative stress on male reproduction. *World Journal of Men’s Health*.

[13] Sheweita S. A., Tilmisany A. M., Al-Sawaf H. (2005). Mechanisms of male infertility: role of antioxidants. *Current Drug Metabolism*.

[14] Bhagavan H. N., Chopra R. K. (2006). Coenzyme Q10: absorption, tissue uptake, metabolism and pharmacokinetics. *Free Radical Research*.

[15] Seren S., Lieberman R., Bayraktar U. D. (2008). Lycopene in cancer prevention and treatment. *American Journal of Therapeutics*.

[16] Zhou X., Liu F., Zhai S. (2007). Effect of L-carnitine and/or L-acetyl-carnitine in nutrition treatment for male infertility: a systematic review. *Asia Pacific Journal of Clinical Nutrition*.

[17] Talevi R., Barbato V., Fiorentino I., Braun S., Longobardi S., Gualtieri R. (2013). Protective effects of in vitro treatment with zinc, d-aspartate and coenzyme q10 on human sperm motility, lipid peroxidation and DNA fragmentation. *Reproductive Biology and Endocrinology: Revista Brasileira de Entomologia*.

[18] Fallah A., Mohammad-Hasani A., Colagar A. H. (2018). Zinc is an essential element for male fertility: a review of Zn roles in men’s health, germination, sperm quality, and fertilization. *Journal of Reproduction and Infertility*.

[19] Ognjanović B. I., Marković S. D., Ethordević N. Z., Trbojević I. S., Stajn A. S., Saicić Z. S. (2010). Cadmium-induced lipid peroxidation and changes in antioxidant defense system in the rat testes: protective role of coenzyme Q(10) and vitamin E. *Reproductive Toxicology*.

[20] Ateşşahin A., Karahan I., Türk G., Gür S., Yilmaz S., Ceribaşi A. O. (2006). Protective role of lycopene on cisplatin-induced changes in sperm characteristics, testicular damage and oxidative stress in rats. *Reproductive Toxicology*.

[21] Dehghani F., Hassanpour A., Poost-Pasand A., Noorafshan A., Karbalay-Doust S. (2013). Protective effects of L-carnitine and homogenized testis tissue on the testis and sperm parameters of busulfan-induced infertile male rats. *Iranian Journal of Reproductive Medicine*.

[22] Dissanayake D., Wijesinghe P., Ratnasooriya W., Wimalasena S. (2009). Effects of zinc supplementation on sexual behavior of male rats. *Journal of Human Reproductive Sciences*.

[23] Adelakun S. A., Ukwenya V. O., Ogunlade B. S., Aniah J. A., Ibiayo A. G. (2019). Nitrite-induced testicular toxicity in rats: therapeutic potential of walnut oil. *JBRA Assisted Reproduction*.

[24] El‐Neweshy M., El‐Maddawy Z., El‐Sayed Y. (2013). Therapeutic effects of date palm (*Phoenix dactylifera* L.) pollen extract on cadmium‐induced testicular toxicity. *Andrologia*.

[25] Mudry M. D., Palermo A. M., Merani M. S., Carballo M. A. (2007). Metronidazole-induced alterations in murine spermatozoa morphology. *Reproductive Toxicology*.

[26] Akhtar M. F., Younas S., Saleem A. (2021). Maternotoxicity and fetotoxicity in *Rattus norvegicus* albinus exposed to tramadol during the late phase of pregnancy. *Birth Defects Research*.

[27] Akhtar M. F., Raza S. A., Saleem A. (2021). Appraisal of anti-arthritic and anti-inflammatory potential of folkloric medicinal plant peganum harmala. *Endocrine, Metabolic & Immune Disorders - Drug Targets*.

[28] Akhtar M. F., Zubair S., Saleem A., Alsharif K. F., Abdel-Daim M. M. (2021). Comparison of individual and combination treatments with naproxen, prednisolone and hydroxychloroquine to treat complete freund’s adjuvant induced arthritis. *Inflammopharmacology*.

[29] Tamilselvan P., Langeswaran K., Revathy R., Kumar B. L., Balasubramanian M. P. (2014). Lycopene, a carotenoid antioxidant against bisphenol a (BPA) motivated experimental male infertility. *Journal of Biomedical and Therapeutic Sciences*.

[30] Nna V. U., Ujah G. A., Mohamed M. (2017). Cadmium chloride–induced testicular toxicity in male wistar rats; prophylactic effect of quercetin, and assessment of testicular recovery following cadmium chloride withdrawal. *Biomedicine & Pharmacotherapy*.

[31] Asadi M. H., Zafari F., Sarveazad A. (2014). Saffron improves epididymal sperm parameters in rats exposed to cadmium. *Nephro-Urology Monthly*.

[32] Elbashir S., Magdi Y., Rashed A., Ibrahim M. A., Edris Y., Abdelaziz A. M. (2018). Relationship between sperm progressive motility and DNA integrity in fertile and infertile men. *Middle East Fertility Society Journal*.

[33] Ibrahim A. T. A. (2015). Protective role of lycopene and vitamin E against diazinon-induced biochemical changes in *Oreochromis niloticus*. *African Journal of Environmental Science and Technology*.

[34] Banihani S., Agarwal A., Sharma R., Bayachou M. (2014). Cryoprotective effect ofl-carnitine on motility, vitality and DNA oxidation of human spermatozoa. *Andrologia*.

[35] Sohrabi M., Hosseini M., Inan S. (2017). Effect of antioxidants on testicular iNOS and eNOS after high-fat diet in rat. *Pakistan Journal of Biological Sciences*.

[36] Çeribaşi A. O., Türk G., Sönmez M., Sakin F., Ateşşahin A. (2010). Toxic effect of cyclophosphamide on sperm morphology, testicular histology and blood oxidant‐antioxidant balance, and protective roles of lycopene and ellagic acid. *Basic and Clinical Pharmacology and Toxicology*.

[37] Azizollahi G., Azizollahi S., Babaei H., Kianinejad M., Baneshi M. R., Nematollahi-mahani S. N. (2013). Effects of supplement therapy on sperm parameters, protamine content and acrosomal integrity of varicocelectomized subjects. *Journal of Assisted Reproduction and Genetics*.

[38] Alharthi W. A., Hamza R. Z., Elmahdi M. M., Abuelzahab H. S. H., Saleh H. (2019). Selenium and L-carnitine ameliorate reproductive toxicity induced by cadmium in male mice. *Biological Trace Element Research*.

[39] Ma J., Han R., Li Y., Cui T., Wang S. (2020). The mechanism of zinc sulfate in improving fertility in obese rats analyzed by sperm proteomic analysis. *BioMed Research International*.

[40] Abd-Allah A. R. A., Helal G. K., Al-Yahya A. A., Aleisa A. M., Al-Rejaie S. S., Al-Bakheet S. A. (2009). Pro-inflammatory and oxidative stress pathways which compromise sperm motility and survival may be altered by L-carnitine. *Oxidative Medicine and Cellular Longevity*.

[41] Chemek M., Venditti M., Boughamoura S., Mimouna S. B., Messaoudi I., Minucci S. (2018). Involvement of testicular DAAM1 expression in zinc protection against cadmium-induced male rat reproductive toxicity. *Journal of Cellular Physiology*.

[42] Meng X., Li L., An H. (2021). Lycopene alleviates titanium dioxide nanoparticle-induced testicular toxicity by inhibiting oxidative stress and apoptosis in mice. *Biological Trace Element Research*.

[43] Xu A., Wang J., Wang H., Sun Y., Hao T. (2019). Protective effect of lycopene on testicular toxicity induced by Benzo[a]pyrene intake in rats. *Toxicology*.

[44] Salem E. A., Salem N. A., Maarouf A. M., Serefoglu E. C., Hellstrom W. J. (2012). Selenium and lycopene attenuate cisplatin-induced testicular toxicity associated with oxidative stress in wistar rats. *Urology*.

[45] Tian Y., Xiao Y., Wang B., Sun C., Tang K., Sun F. (2018). Vitamin E and lycopene reduce coal burning fluorosis-induced spermatogenic cell apoptosis via oxidative stress-mediated JNK and ERK signaling pathways. *Bioscience Reports*.

[46] Abd‐Elrazek A., Ahmed‐Farid O. (2018). Protective effect of L‐carnitine and L‐arginine against busulfan‐induced oligospermia in adult rat. *Andrologia*.

[47] Khedr N. F., Werida R. (2021). L-carnitine modulates autophagy, oxidative stress and inflammation in trazodone induced testicular toxicity. *Life Sciences*.

[48] Dokmeci D., Inan M., Basaran U. N. (2007). Protective effect of L-carnitine on testicular ischaemia-reperfusion injury in rats. *Cell Biochemistry and Function*.

[49] Soliman M. M., Elshazly S. A., Aldhahrani A. (2020). Gamma‐irradiation‐induced testicular oxidative stress and apoptosis: mitigation by L‐carnitine. *Journal of Biochemical and Molecular Toxicology*.

[50] Sainao W.-Q., Zhang M. D., Ma X. J., Ran R. L., Jia L. Y., Feng H. Q. (2018). Physiological effects of cadmium stress on Astragalus membranaceus seedlings and alleviative effects of attapulgite clay on cadmium stress. *Zhongguo Zhong Yao Za zhi*.

